# Gambling problems in the family – A stratified probability sample study of prevalence and reported consequences

**DOI:** 10.1186/1471-2458-8-412

**Published:** 2008-12-16

**Authors:** Hanne Gro Wenzel, Anita Øren, Inger Johanne Bakken

**Affiliations:** 1 Division of Psychiatry, Orkdal Department, St Olav University Hospital, Norway; 2 Department of Epidemiology, SINTEF Health Research, Abels gate 5, 7465-Trondheim, Norway

## Abstract

**Background:**

Prior studies on the impact of problem gambling in the family mainly include help-seeking populations with small numbers of participants. The objective of the present stratified probability sample study was to explore the epidemiology of problem gambling in the family in the general population.

**Methods:**

Men and women 16–74 years-old randomly selected from the Norwegian national population database received an invitation to participate in this postal questionnaire study. The response rate was 36.1% (3,483/9,638). Given the lack of validated criteria, two survey questions ("Have you ever noticed that a close relative spent more and more money on gambling?" and "Have you ever experienced that a close relative lied to you about how much he/she gambles?") were extrapolated from the Lie/Bet Screen for pathological gambling. Respondents answering "yes" to both questions were defined as Concerned Significant Others (CSOs).

**Results:**

Overall, 2.0% of the study population was defined as CSOs. Young age, female gender, and divorced marital status were factors positively associated with being a CSO. CSOs often reported to have experienced conflicts in the family related to gambling, worsening of the family's financial situation, and impaired mental and physical health.

**Conclusion:**

Problematic gambling behaviour not only affects the gambling individual but also has a strong impact on the quality of life of family members.

## Background

Pathological gambling affects the gambler's closest family and financial problems are often encountered [1]. There may be a loss of money to pay for essentials, huge credit card debts, and illegal loans, loss of rent money, and eviction [2,3]. Life in a problem gambler's family is often beset with crises and conflicts and distress levels are elevated [1]. In their studies on problem gambling in the family, Hodgins and co-workers utilize the term "Concerned Significant Others" (CSOs) [4,5], which we will also employ. In a sample of CSOs, the Global Severity Index, a measure of general distress, was significantly increased compared to normative scores for adult non-patients, but lower than scores for adult mental health outpatients [4]. The emotional climate in the problem gambler's family is typically characterized by distrust and insecurity, and emotional and psychosomatic difficulties are common. In a study among Gam-Anon members, the family component of Gamblers Anonymous, more than four in five respondents reported that their experiences had caused them emotional illness [6]. High levels of emotional, verbal, and physical abuse and of suicide attempts have been observed among family members of problem gamblers [3,7]. In attempt to cope with the situation spouses and family members may resort to dysfunctional patterns of behavior with excessive drinking, smoking, or overeating [3].

The aims of the present probability sample study were to estimate the proportion of problem gambler's CSOs in the Norwegian population. We hypothesized that problem gambling in the family might lead to family conflicts and could have impact on CSO health.

## Methods

The data in the current study were collected as part of a national postal gambling survey that also included a range of questions on gambling in the family. A total of 10,000 individuals were drawn from the national population database in a random procedure which was stratified by age, gender, and county of residence. The study questionnaire and a pre-paid envelope were sent in January 2007, with a reminder to non-respondents two months later. As an alternative to the paper questionnaire, respondents could answer the questions at the study web site. Access to the web site was possible by the use of a personal code sent to each individual. The final data file was checked for duplicates. For individuals who completed the postal questionnaire and also accessed the study web site, only answers from the postal questionnaire were included to the database. Three-hundred-and-sixty-two invitations were returned as "address unknown", reducing the population sample to 9,638 individuals. In total, 3,483 individuals completed the questionnaire, which gave a response rate of 36.1% (3,483/9,638). The achieved sample consisted of 53.1% women and 46.9% men (issued sample: 48.9% and 51.1%, respectively). The age distribution of the achieved sample (figures for issued sample reported in parentheses) was as follows: 16–20 years: 4.9% (9.4%), 21–25 years: 4.7% (8.3%), 26–45 years: 39.4% (39.3%), 46–55 years: 22.8% (18.5%), 56–65 years: 20.0% (16.0%), 66–74 years: 8.1% (8.4%).

The two central questions used to assess problem gambling in the family were adapted from the Lie/Bet Screen [8,9]. The Lie/Bet Screen was derived from the DSM-IV criteria and is a 2-item screen for pathological gambling ("Have you ever felt the need to bet more and more money?" and "Have you ever had to lie to people important to you about how much you gamble?"). The adapted questions used in the present study were "Have you ever noticed that a close relative spent more and more money on gambling?" and "Have you ever experienced that a close relative lied to you about how much he/she gambles?" Respondents answering "yes" to both questions were defined as CSOs to problem gamblers. All other respondents were defined as non-CSOs. The two original Lie/Bet questions were used to assess the respondent's own gambling problems. Respondents answering "yes" to both questions were classified as having gambling problems.

We applied a post-stratification probability sampling method weighted on gender, age group, and residence (county) between the achieved sample and population estimates. Educational level was categorized as low (no high school), medium (high school), and high (beyond high school), according to national recommendations [10]. Chi-square tests were used for linear trend analysis. All other analyses were carried out in the software Statistical Package for the Social Sciences (SPSS) for Windows version 14.0 (SPSS, Chicago, IL). We used log-likelihood tests for univariate and multivariate analyses. Variables significantly associated with being a CSO were identified using multivariate logistic regression analyses, with variables with a P ≤ 0.10 in univariate analysis as input to the model. Final variable selection was based on the forward-stepwise method.

The study was approved by the Norwegian Data Inspectorate and the Regional Committee for Medical Research Ethics, Central Norway.

## Results

In the total sample, 3.4% (118/3,482) of respondents reported to have ever noticed that a close relative spent more and more money on gambling, while 2.9% (100/3,482) reported to have ever experienced that a close relative lied about the amount of gambling.

The overall proportion of CSOs (both questions answered positively) was 2.0% (70/3,482) (Table 1). In univariate analyses, female gender, young age, living in a city, being divorced, being in an unsatisfactory financial situation, and having an unsatisfactory subjective health were all variables significantly associated with being a CSO. In multivariate analysis including all variables significant in univariate analysis, gender and financial situation remained the only significant variables (Table 1, Model 1). As an unsatisfactory financial situation and an unsatisfactory subjective health might be caused by gambling problems in the family, we also carried out an alternative analysis, where financial situation and health were omitted from the model (Table 1, Model 2). In this model the variables gender, age, and marital status were all significant.

**Table 1:** Proportion of Concerned Significant Others (CSOs) to problem gamblers and variables associated with being a CSO

	N (% in sample)	Proportion (%) of CSOs (95% CI)	Unadjusted odds ratio (95% CI)	Model 1* Adjusted odds ratio (95% CI)	Model 2^§ ^Adjusted odds ratio (95% CI)
*All respondents*	3,482 (100)	2.0 (1.6–2.5)			
*Gender*					
Female	1,716 (49.3)	3.0 (2.2–3.9)	2.8 (1.6–4.7)	2.7 (1.6–4.6)	2.6 (1.5–4.4)
Male (ref)	1,766 (50.7)	1.1 (0.7–1.7)	1	1	1
*Age (years)*					
16–24	538 (15.5)	2.7 (1.6–4.4)	2.1 (1.1–4.2)	-	2.4 (1.1–5.5)
25–44	1,346 (38.7)	2.6 (1.9–3.6)	2.1 (1.2–3.6)	-	2.1 (1.2–3.7)
45–74 (ref)	1,598 (45.9)	1.3 (0.8–2.0)	1	-	1
*Domicile*					
City	1,747 (50.2)	2.4 (1.8–3.2)	2.3 (1.1–4.7)	-	-
Small town	879 (25.2)	2.2 (1.4–3.4)	2.0 (0.9–4.6)	-	-
Countryside (ref)	787 (22.6)	1.1 (0.6–2.1)	1	-	-
*Marital status*					
Married, cohabitating, widow/widower (ref)	2,431 (69.8)	1.7 (1.3–2.3)	1	-	1
Single/Never married	770 (22.1)	2.2 (1.4–3.5)	1.3 (0.7–2.3)	-	1.0 (0.5–1.9)
Divorced/Separated	259 (7.4)	4.0 (2.2–7.1)	2.3 (1.2–4.7)	-	2.6 (1.3–5.2)
*Financial situation*					
Good (ref)	2,274 (65.3)	1.3 (0.9–1.8)	1	1	-
Average	928 (26.7)	3.1 (2.2–4.4)	2.5 (1.5–4.2)	2.4 (1.4–4.1)	-
Unsatisfactory	247 (7.1)	4.9 (2.8–8.4)	4.0 (2.0–8.0)	3.9 (2.0–7.9)	-
*Subjective Health*					
Good (ref)	2,563 (73.6)	1.8 (1.4–2.4)	1	-	-
Average	722 (20.7)	2.1 (1.3–3.4)	1.2 (0.6–2.1)	-	-
Unsatisfactory	174 (5.0)	5.1 (2.7–9.4)	2.9 (1.4–6.1)	-	-

*Model 1: All variables with P = 0.10 in univariate logistic regression entered to multivariate logistic regression analyses

^§^Model 2: The variables financial situation and subjective health were omitted from multivariate logistic regression analyses

Respondents were asked in what ways gambling in the family had affected their lives (Table 2). Compared to non-CSOs, CSOs significantly more often reported that gambling in the family had led to worsening of family finances, isolation from friends and family, conflicts in the family, reduced mental health, or reduced physical health.

**Table 2:** Reported effects* of gambling in the family among Concerned Significant Others (CSOs) and non-CSOs

	Non-CSOs (%)	CSOs (%)	
N	3,412	70	P-value^§^
Improvement of family financial situation	1.4	1.4	-
Worsening of family financial situation	1.0	46.3	< 0.001
Less contact with family and friends	0.2	8.7	< 0.001
Conflicts in the family	1.2	64.9	< 0.001
Reduced mental health (anxiety, depression)	0.3	16.6	< 0.001
Reduced physical health (muscle tension, headache, stomach ache etc)	0.3	17.8	< 0.001

* Respondents were asked to answer the question: "What has it meant to you that someone in your family is gambling/has been gambling?" (multiple response question)

^§ ^From Chi-square

Table 3 shows that CSOs significantly more often than non-CSOs reported that they had sleep disorders, had experienced depression/feeling down, or defined themselves as having obsession/compulsions or as alcohol/substance abusers. Finally, Table 3 shows that the frequency of own gambling problems was higher among CSOs than among non-CSOs.

**Table 3:** Self-reported mental health problems (%) among Concerned Significant Others (CSOs) and non-CSOs

	Non-CSOs	CSOs	
N	n = 3,412	n = 70	P-value*
Sleep disorders	27.0	38.8	0.032
Symptoms of depression/feeling down	16.8	35.0	< 0.001
Suicidal ideations	1.4	0.0	-
Anxiety symptoms	6.6	7.4	-
Obsessive/compulsion symptoms	1.9	5.0	0.026
Alcohol and substance abuse	1.8	9.3	< 0.001
Problem gambling^§^	1.5	8.6	< 0.001

* From Chi-square

^§ ^By the Lie/Bet Screen

## Discussion

The present study shows that gambling problems in the family may have considerable consequences for problem gamblers' CSOs. Two percent of the sample were CSOs by our definition, with the highest prevalences among divorced females below the age of 45. The consequences reported were considerable with almost two thirds of the CSOs reporting family conflicts and almost half of the CSOs indicating worsening of the family financial situation. Both mental health and physical health seemed to be affected by the gambling problems in the family.

To the best of our knowledge, the current epidemiological study is the first to focus on relatives to problem gamblers and their physical and mental health in a random population sample. A recent review of the available literature on gambling problems in the family showed that prior studies in general are small and most often include help-seeking populations [1]. Thus, the major strength of the present study is its population-based cross-sectional design. The study's major weakness is the low response rate (36.1%). The number of respondents was however quite high (3,483) and we adjusted for differences in gender, age-group, and residence (county) between the achieved sample and population estimates by applying a non-response post-stratification weight. The response rate was highest among women 45 years and older (data not shown). The study was an anonymous postal survey and it seems unlikely that respondents with gambling problems in the family would be afraid of stigmatization and therefore refuse to answer. We would rather assume that CSOs were keen responders with a wish for expressing concern with the family's problematic situation. Another limitation of the study is the instrument used to assess problem gambling in the family. As there are no validated CSO criteria, we used questions adapted from a screen for assessment of problem gambling, the Lie/Bet Screen. This screen has been validated internationally and also in a Norwegian population [8,9]. Validation of the adapted screen would however require matching between information from CSOs and their relatives. Such validation was not possible based on the data available and the prevalences in the present study should be regarded as estimates only. The data in the current study were collected as part of a large national survey of gambling behaviour and problem gambling. The length of the questionnaire restricted us from including clinically validated instruments for the assessment of psychological impairments, which is another important limitation of our study. However, self-reported health has been shown to be an important health indicator in population studies [11].

We found a slightly higher prevalence of family gambling problems as indicated by relatives (2.0%) compared to the prevalence of problem gambling indicated by the gamblers themselves (1.6%), which seems reasonable because on average, each problem gambler's behavior is likely to affect more than one family member. Our findings indicate that CSOs have a high awareness of family gambling problems and may therefore constitute a way to reach the family with counselling and treatment approaches, especially in cases when problem gamblers deny their situation and are resistant to seek help.

The respondents did not report their particular relationship to the gambler. However, the majority of CSOs were female and we find it likely that they mainly were spouses or former spouses. This assumption is supported both by our study and prior studies. The present study showed that being young, female and divorced or separated were all factors significantly associated with being a CSO. Correspondingly, elevated divorce rates among problem gamblers have been found in both clinical studies and surveys [1]. In our data, conflicts in the family related to gambling were reported by two-thirds of CSOs. Although somewhat speculative, data in the current study held together with prior findings indicate that gambling in many cases might be a direct reason for divorce.

We found that one in six CSOs reported a reduction in their mental health that they directly related to gambling in the family, and a similar proportion reported that gambling in the family had led to impaired physical health. Furthermore, CSOs more often reported mental health problems in general (sleep disorders, feeling of depression) compared to non-CSOs. In a study among female Gam-Anon members, four in five respondents reported to experience physical symptoms such as headache, breath irregularities, irritable bowel, and dizziness during periods with intense gambling, while mental symptoms such as anger, guilt, confusion, hopelessness, and depression were reported by three in four [7]. Several studies have indicated that such health problems should be seen as a consequence of the chronic stress experienced through living with a problem gambler rather than a personal defect in the CSOs [1,3,12,13]. Consequently, recent research has increasingly focused on coping skills training programs as treatment for health problems in relatives to problem gamblers [5,13,14]. Lorenz has commented that the coping skills in CSOs seem to be inadequate and in several cases self-destructive with isolation, self-abuse behavior and alcoholism [3,7], which corresponds to our findings that CSOs reported conflicts in the family and reduced contact with family and friends as direct consequences of gambling, and also more often reported obsession/compulsions and alcohol/substance abuse compared to non-CSOs. Our results show that CSOs often are in need of counseling and help because of the problems they encounter, which is in concordance with the Gam-Anon study, where three in four members stated that they could have benefited from mental health counseling [7]. The elevated prevalence of own gambling problems as measured by the Lie/Bet Screen among CSOs should also be remarked. Concentration of gambling problems within families will obviously aggravate the situation with more financial problems and decreased family resources for coping.

## Conclusion

Our study shows that CSOs to problem gamblers encounter widespread effects including both physical symptoms and emotional distress. Further research should focus on prospective studies were data are collected both during periods with intense gambling and during periods with abstinence. Evidence-based counseling and treatment approaches to meet the needs of problem gamblers' families are clearly needed.

## Competing interests

The authors declare that they have no competing interests.

## Authors' contributions

AØ conceived of the study and coordinated data collection. HGW and IJB contributed equally in the writing of the initial manuscript draft and subsequent revisions. IJB performed the statistical analyses. All authors contributed to study design and have read and approved the final manuscript.

## Pre-publication history

The pre-publication history for this paper can be accessed here:


